# RNA Sequencing in COVID-19 patients identifies neutrophil activation biomarkers as a promising diagnostic platform for infections

**DOI:** 10.1371/journal.pone.0261679

**Published:** 2022-01-26

**Authors:** Richard Wargodsky, Philip Dela Cruz, John LaFleur, David Yamane, Justin Sungmin Kim, Ivy Benjenk, Eric Heinz, Obinna Ome Irondi, Katherine Farrar, Ian Toma, Tristan Jordan, Jennifer Goldman, Timothy A. McCaffrey

**Affiliations:** 1 Department of Medicine, Division of Genomic Medicine, The George Washington University Medical Center, Washington, DC, United States of America; 2 Department Anesthesiology and Critical Care Medicine, The George Washington University Medical Center, Washington, DC, United States of America; 3 Department of Emergency Medicine, The George Washington University Medical Center, Washington, DC, United States of America; 4 Department of Clinical Research and Leadership The George Washington University Medical Center, Washington, DC, United States of America; 5 True Bearing Diagnostics, Washington, DC, United States of America; 6 Department of Microbiology, Immunology, and Tropical Medicine, The George Washington University Medical Center, Washington, DC, United States of America; Hamad Medical Corporation, QATAR

## Abstract

Infection with the SARS-CoV2 virus can vary from asymptomatic, or flu-like with moderate disease, up to critically severe. Severe disease, termed COVID-19, involves acute respiratory deterioration that is frequently fatal. To understand the highly variable presentation, and identify biomarkers for disease severity, blood RNA from COVID-19 patient in an intensive care unit was analyzed by whole transcriptome RNA sequencing. Both SARS-CoV2 infection and the severity of COVID-19 syndrome were associated with up to 25-fold increased expression of neutrophil-related transcripts, such as neutrophil defensin 1 (DEFA1), and 3-5-fold reductions in T cell related transcripts such as the T cell receptor (TCR). The DEFA1 RNA level detected SARS-CoV2 viremia with 95.5% sensitivity, when viremia was measured by ddPCR of whole blood RNA. Purified CD15+ neutrophils from COVID-19 patients were increased in abundance and showed striking increases in nuclear DNA staining by DAPI. Concurrently, they showed >10-fold higher elastase activity than normal controls, and correcting for their increased abundance, still showed 5-fold higher elastase activity per cell. Despite higher CD15+ neutrophil elastase activity, elastase activity was extremely low in plasma from the same patients. Collectively, the data supports the model that increased neutrophil and decreased T cell activity is associated with increased COVID-19 severity, and suggests that blood DEFA1 RNA levels and neutrophil elastase activity, both involved in neutrophil extracellular traps (NETs), may be informative biomarkers of host immune activity after viral infection.

## Introduction

The COVID-19 pandemic, initiated by SARS-CoV2 viral infection, illustrates the need for the rapid detection of novel and variant pathogens, as well as specific risk prediction models to identify patients at high risk of severe or fatal disease. Unfortunately, clinical risk models for critically ill patients have been only modestly accurate in predicting outcomes for COVID-19 patients [1]. Furthermore, similar to septic shock, there is an incomplete understanding of the relative contributions of the pathogen load versus a hyperactive immune response by the host. The detection of SARS-CoV2 viral load has mostly been measured only in the accessible regions of the respiratory tract, and requires prior knowledge of the viral sequence to achieve relatively specific PCR amplification. The current studies investigated a complementary strategy that utilizes the host immune system to report a pathogen’s presence and activity. Truly ‘agnostic’ blood biomarkers of infection would have numerous uses, especially in helping to discriminate infectious diseases from aseptic inflammation, such as might occur in asthma, allergies, fibrotic, or autoimmune disorders.

In previous work, we employed whole transcriptome profiling to identify RNA biomarkers in peripheral blood from patients with appendicitis or respiratory infections [2]. Relatively large fold-change increases (~20-fold) were identified in RNAs coding for neutrophil primary granule markers, such as neutrophil defensins (DEFA1), in infections where the pathogen was directly accessible to the immune system, such as pneumonia. In appendicitis, where the infection can take the form of a ‘biofilm’ that is not directly accessible to the immune cells [3], there was marked increases in mRNAs coding for IL8 receptor-ß (IL8RB/CXCR2), and secondary granule proteins such as alkaline phosphatase (ALPL) [2]. Thus, it was hypothesized that the RNA biomarkers would report immune activation in response to a SARS-CoV2 infection, regardless of the virus strain, and provide a measure of the activation of the host immune response.

A general limitation to using RNA-based tests, however, is that accurately measuring blood RNA biomarkers in a clinical setting has been hampered by the instability of blood RNA and the complexity of RNA purification and quantitation. Thus, RNA biomarkers, while sensitive and accurate, are presently too slow to be applied in any acute clinical setting. Conversely, using circulating protein biomarkers of infection or sepsis is technically more feasible in the clinic, but sensitivity and accuracy of most current measures are marginal. While circulating markers, such as C-reactive protein (CRP) and lactate, are currently in clinical use, they have relatively weak predictive power relative to potentially life-threatening outcomes such as sepsis [4]. While many promising markers have been identified, their use in the clinic have been hampered by the complexity of blood and its innate ability to bind or degrade inflammatory cell products.

While COVID-19 is principally initiated and driven by SARS-CoV2 virus, next generation sequencing proves that secondary bacterial and fungal infections are readily detected in COVID-19 cases. Secondary infections in COVID-19 patients have been detected in the range of 25–50% of patients, with a predominance of Klebsiella and Staphylococcus [5, 6]. The severity of COVID-19 syndrome has been consistently shown to be related to an increase in the neutrophil/lymphocyte ratio (NLR) [7, 8]. Severe COVID-19 is thought to result from a ‘cytokine storm’ devolving into intrapulmonary and intravascular neutrophil extracellular traps (NETs) that compromise oxygen exchange [9–12]. Thus, while neutrophil activation might represent a useful strategy for detecting infection and the host response, a practical method at the point of care is lacking. Furthermore, purification of neutrophils from human blood by conventional density methods for RNA or functional analysis is time-consuming and typically requires centrifugation that can yield activated or damaged cells.

Thus, a powerful addition to the diagnostic arsenal would be a test that detects novel or known pathogens through the activation of the host immune response. The present studies employed RNA sequencing (RNAseq) to identify blood RNA biomarkers of COVID-19 infection and severity, and suggested the use of NET-related factors as alternative biomarkers that could be measured rapidly and inexpensively at the point of care.

## Materials and methods

### Enrollment and blood draw

This prospective, observational study was approved by the Institutional Review Board of The George Washington University (#NCR202539). All subjects, or their legally authorized surrogate, provided informed consent. Patients admitted into The George Washington University Hospital Intensive Care Unit were screened for inclusion and exclusion criteria. Inclusion criteria consisted of age greater than or equal to 18 and the presence of a positive SARS-CoV2 test. Normal controls were sampled from asymptomatic medical center personnel known or assumed to be SARS-CoV2 negative. After informed consent was received, venipuncture, or sampling from an indwelling catheter, was performed to draw whole blood in Tempus Blood RNA tubes (ThermoFisher) and BD Vacutainer K2 EDTA tubes (BD Biosciences). Researchers who were processing the blood samples and performing assays were blinded to the subjects’ clinical characteristics and outcomes by anonymous coding of the samples.

### Neutrophil isolation with anti-CD15+ magnetic beads

Within 20 minutes post-collection, the CD15+ cells (comprising neutrophils, scarce eosinophils, and a rare subtype of T cells) were isolated from the EDTA-treated blood sample using anti-human CD15 antibody-coated Dynabeads (Invitrogen) with minor modifications to the manufacturer’s protocol. Immediately before use, the beads were re-suspended and washed as recommended. The bead isolation buffer (BIB) was modified by using HBSS without calcium or magnesium (Gibco). Blood volumes of 750 μl were used in 1.5 mL tubes. When larger volumes were processed, multiples of 750 μl were used. Instead of the recommended initial wash steps, the blood was spun at 3000 rpm (604 g) for 10 minutes, the plasma removed, and 750 μl of BIB was added just before adding the beads. Each wash step used a 1:1 volume to the original blood (typically 750 μl BIB), to keep a 1x concentration of the CD15+ cells. Cell capture and washing were performed using the MAGNA-SEP magnet (Invitrogen) for two minutes. Cell counts of the 1x isolate were performed using 10 μl in a hemocytometer at 200X using phase contrast. The identities of the isolated cells were confirmed by fixing with 4% formaldehyde in 1x PBS, and using a May-Grünwald stain (Wright-Giemsa stain with eosin Y).

### Whole blood cell count and CD15+ yield calculation

For both white blood cell counts (WBC) and differential counts, we used the same blood as for the isolation of the CD15+ cells and they were performed simultaneously with the isolation. WBC counts were achieved by adding 20 μl of whole blood to a Leukotic tube (Bioanalytic), following the product’s protocol, ensuring the cells remained homogenously suspended, and counting the preserved cells within 2 hours in a hemocytometer at 200X. Whole blood smears were performed in triplicate. Differential staining was achieved by fixation in methanol (10 min; ThermoFisher Scientific), eosin Y staining (40 s; Sigma Aldrich), Wright Giemsa staining (100% 1:30 min, then 50% 3 min; VWR), and finishing with Permount (Electron Microscopy Sciences) and a coverslip. For each sample, 200 or more cells were counted within the feathered edges of the smears. CD15+ yield calculations were performed by comparing the isolate’s cell count to the WBC’s fraction of neutrophils and eosinophils as determined by the differential count.

### Whole blood RNA extraction

The Tempus Blood RNA preservation tubes (Applied Biosystems) were stored at -80° C and thawed in a 37° C water bath. RNA was purified using the Tempus Spin RNA Isolation Reagent Kit (Applied Biosystems). The Tempus blood lysate was spun at 3000 g for 30 minutes, and the pellet was then resuspended and transferred onto purification columns. Following on-column DNAse treatment using a TurboDNase kit (Ambion) and a series of washing and drying steps, purified RNA was eluted with nuclease-free water. RNA levels were quantified using the NanoDrop ND-1000 Spectrophotometer and quality was determined by capillary electrophoresis on the Agilent 2100 Bioanalyzer.

### RNA biomarker digital droplet PCR (ddPCR)

Primers and probes for DEFA1, IL8RB, ALPL, RSTN, MPO, and ACTB were designed using Geneious Prime software and were synthesized by Biosynthesis. RNA (200 ng) was reverse transcribed into cDNA using gene-specific primers (250 nM) with the RNAse H+ reverse transcriptase and 5X Supermix from the iScript Select cDNA Synthesis Kit (Bio Rad). The cDNA was diluted 1/20 in nuclease-free water and placed in a ddPCR 96-well plate (5 μl per well). Each of the markers were paired to make a total of three primer-probe master mixes (4 μM forward primer; 4 μM reverse primer, 1.12 μM probe) with water and the ddPCR Supermix (Bio Rad); 17 μl were added per ddPCR well.

The BioRad Automated Droplet Generator produced at least 10,000 nanoliter-sized droplets per well, the target transcripts were amplified in each droplet by PCR, and each droplet was read separately by the Bio Rad QX200 Droplet Reader. Bio Rad Quantasoft software was used to set the threshold for positive droplets. Copy numbers were normalized by dividing by the number of ACTB copies; this was performed to counter variation in the numbers of cells captured and possible variation in RNA yield efficiency.

### SARS-CoV2 nucleocapsid RNA ddPCR

The Bio-Rad SARS-CoV-2 ddPCR Kit (FDA-cleared for COVID diagnosis from nasal swab RNA) was used to measure nucleocapsid transcripts (N1 and N2) of the SARS-CoV2 in Tempus whole blood RNA. cDNA synthesis and ddPCR was accomplished with a one-step RT-ddPCR mix (Bio Rad) added directly to the RNA. A mix of primers and FAM/HEX probes for N1, N2 and human RNase P (RP) was added (Bio Rad). After droplet generation, the thermal cycler reverse transcribed the RNA and then amplified the cDNA. Droplets were read using the Bio Rad QX200 and clusters were gated using the Bio Rad Quantasoft Analysis Pro software. RP was included as a control to confirm proper amplification and reading. Samples generating two or more N1+ or N2+ droplets were considered positive as specified by the manufacturer.

### RNAseq methods

Whole blood RNA was extracted and DNAsed using the Tempus system described above. Quantity and quality were assessed using a NanoDrop for OD at 260/280 and an Agilent 2100 Bioanalyzer. Each sample had a RIN >8. The RNA (200 ng) was depleted of ribosomal RNA, converted to cDNA, tagged for multiplexing, and amplified using the Illumina TruSeq Stranded Total RNAseq kit. After quality control adjustments, the multiplexed cDNA library was sequenced on an Illumina NextSeq using 24 samples per run with the Illumina TotalRNA labeling and amplification kit.

#### Alignment and transcript counting

The raw files containing 150-bp paired-end reads were concatenated across the 4 sequencing lanes, quality-filtered and trimmed using trimmomatic in the Galaxy software suite. Trimmed reads were aligned to the human hg38 genome (UCSC 197K transcripts) using HiSat2, and then parsed into known transcripts using featureCount. The average yield of aligned paired-end reads was 12.4 million per sample.

#### Bioinformatic analysis

The raw read counts per transcript and transcript sizes were transferred to Excel, where the RPKM was computed (reads per thousand bases of transcript, per million total reads, per patient). The RPKM per transcript per patient was loaded into GeneSpring GX14 to analyze for differentially expressed genes (DEGs). The 197K transcripts were reduced to the gene level (~44K transcripts) and then the DEGs were identified by a triple-filter strategy that excludes transcripts of low expression (<0.01 RPKM in >70% of samples), and then seeks transcripts with a greater than 2-fold change in the geometric mean expression with a t-test p-value of <0.001 (uncorrected for multiple testing). This approach has been shown to be accurate in identifying spiked ‘true’ DEGs in prior studies [13]. Gene ontologies were analyzed with NIH David, and pathways analyzed with Ingenuity Pathway Analysis.

#### Cell type-specific gene expression

Pre-curated transcripts from the Human Blood Atlas identified by RNAseq of purified blood cell types [14] were matched to gene-level transcripts in the RNAseq data set. The average level of expression among detectable transcripts (non-zero in at least one group) was computed and used as an index of cell-type specific expression.

### Elastase assay

CD15+ neutrophils for the fluorometric elastase assay were isolated as described above. Aliquots of 50 μl containing 100-600K cells were stored at -80° C. The elastase assay was performed in batches of 4–16 samples. Each batch was thawed and underwent 5 cycles of freeze/thaw using dry ice in ethanol and a 37° C water bath. Thawed lysates were diluted with 140 μl of PBS without calcium or magnesium and 10 μl of the fluorometric substrate, bis(N-benzyloxycarbonyl-L-tetra-alanyl)rhodamine, (CBZ-Ala-Ala-Ala-Ala)2Rh110, (Anaspec), dissolved in 90% DMSO to 2000 μM. Proteolytic cleavage of the substrate de-quenches the Rhodamine 110 fluorochrome. At specified times, the magnetic beads were removed from the light path with a strong neodymium magnet, and fluorescence intensity was measured on a Qubit 2.0 fluorometer (ThermoFisher Scientific) with 470 nm excitation and 520 nm emission. Mixing and measurements were repeated every 20 minutes for 2 hours.

## Results and discussion

### Clinical characteristics of COVID-19 patients in ICU

Patients were consented and blood was drawn upon admission to the intensive care unit (ICU) near the peak of the COVID-19 pandemic, from November 2020 through March 2021. Because the ICU was used to isolate any suspected COVID-19 cases, they reflected a range of cases from PCR+ but asymptomatic, through moderate to severe COVID-19 symptoms (n = 38). For biomarker analysis, they were divided into 3 groups: 1) Incidental patients (Incidental, n = 7), were SARS-CoV2 PCR+ in nasal swabs, but their COVID-like symptoms were mild or unnoticeable, and were not the reason for admission to the hospital/ICU. These patients were admitted for a range of reasons including trauma and elective surgery. 2) Moderate COVID patients (Moderate, n = 7) were admitted for symptoms consistent with COVID-19 and tested PCR+ by nasal swab, but did not require intubation or vasopressors. 3) Severe COVID-19 patients were PCR+ by nasal swab and met at least 1 of 3 criteria: intubation, vasopressors, or fatality (Critical, n = 24).

The patient characteristics are consistent with prior observations that more severe cases of COVID-19 are associated with increasing age and BMI (Table 1). There were not striking associations of severity with comorbid conditions such as Type II diabetes or hypertension (Table 1), or other comorbidities such as cardiovascular or pulmonary disease, although it is important to note that the present study was not designed, nor powered to detect those differences (S1 Table). Of note, there was a considerable discrepancy between the clinical classification of the patients, based on nasal swab PCR for SARS-CoV2 and symptoms, versus the laboratory measures of SARS-CoV2 viral titer in blood as determined by ddPCR. In short, 2 of the 7 Incidental patients, and one of the Moderate patients actually had very high titers of SARS-CoV2 in blood, which obscured the predicted differences in blood titers between groups (Table 1).

**Table 1:** Clinical characteristics of ICU patients included in the study.

	Incidental		Moderate		Critical		
Demographics	Mean	S.E.M.	Mean	S.E.M.	Mean	S.E.M.	
**N per group**	**7**		**7**		**24**		
**Sex (% Male)**	**57.1%**		**42.9%**		**45.8%**		
**Age (years)**	**54.0**	6.9	**46.7**	3.2	**62.1**	1.7	*
**BMI**	**30.4**	2.7	**37.3**	4.1	**32.8**	1.9	
**African or African-American**	**42.9%**		**85.7%**		**58.3%**		
**Co-Morbidity**							
**T2DM (Type II Diabetes Mellitus)**	**42.9%**		**42.9%**		**50.0%**		
**HTN (Hypertension)**	**71.4%**		**57.1%**		**66.7%**		
**Treatments**							
**Intubation**	**14.3%**		**0.0%**		**79.2%**		*
**BiPAP**	**0.0%**		**42.9%**		**12.5%**		
**ECMO VV**	**0.0%**		**0.0%**		**8.3%**		
**Dexamethasone**	**57.1%**		**100.0%**		**83.3%**		
**Vasopressor**	**0.0%**		**0.0%**		**66.7%**		*
**Clinical Measures**							
**PF Ratio**	**2.51**	**0.95**	**1.41**	**0.42**	**1.76**	**0.26**	
**Fatality**	**14.3%**		**0.0%**		**50.0%**		*
**Laboratory Results**							
**SARS-CoV2 blood titer (ddPCR)**	**1068.77**	969.39	**1063.36**	1094.9	**478.3**	272.63	
**WBC Count**	**11.99**	3.43	**10.03**	2.43	**14.98**	1.37	
**Lymphocyte Count**	**1.18**	0.36	**1.13**	0.26	**1.05**	0.17	
**Neutrophil Count**	**9.65**	2.23	**9.88**	2.41	**14.05**	1.32	
**Neutrophil/Lymphocyte Ratio (NLR)**	**10.23**	2.88	**11.93**	5.13	**25.38**	4.69	
**Platelet Count**	**308.71**	65.9	**223.14**	37.05	**273.82**	26.19	
**Hemoglobin**	**11.13**	1.17	**12.39**	0.99	**9.38**	0.47	*
**Yield (ug) from 3 mL blood**	**9.3**	3.1	**10.8**	2.8	**15.0**	2.4	

### RNA sequencing of blood RNA from COVID-19 patients

**Fig 1:**
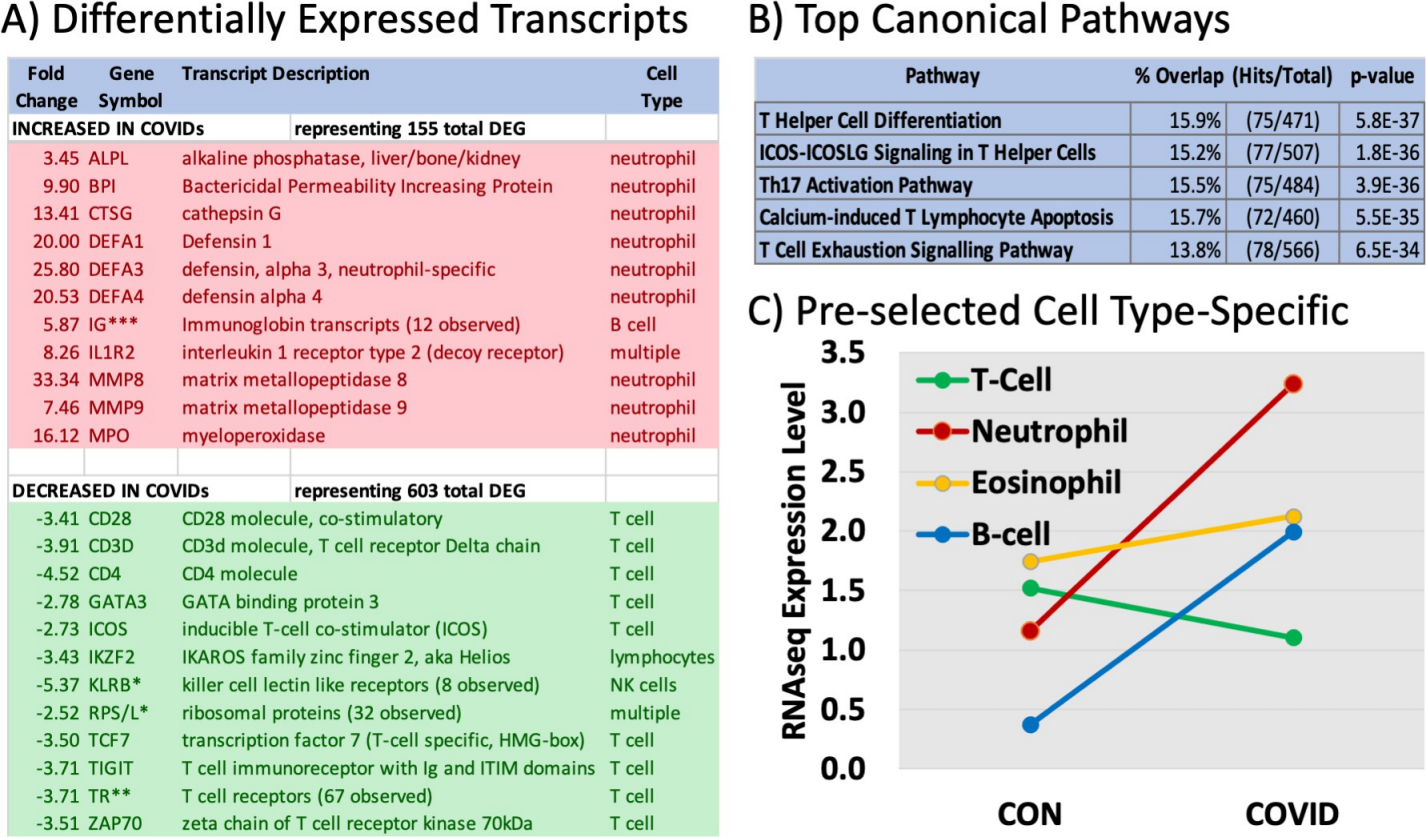
Cell type-specific and differentially expressed genes (DEGs) between COVID-19 and controls. ***Panel A*:** The RNAseq data was analyzed to identify differentially expressed genes (DEGs) between controls/incidentals (7) and COVID-19 patients (17) using a triple filtering approach that excluded transcripts with low absolute levels (<0.01 RPKM), and then testing for an absolute fold-change of >2 with a t-test p-value of less than 0.001 uncorrected. A representative subset is shown for increased (red) vs decreased (green) transcripts in COVID-19 patients. ***Panel B*:** The total list of 758 DEGs was analysed for the pathways affected using IPA. The 5 top pathways are shown with the % of overlap to the precurated list, the number of transcript matches, and the p-value of the overlap. ***Panel C*:** Cell-type-specific RNAs (~10 per cell type) were extracted from the Blood Atlas and then their levels were computed from the RNAseq data of control vs COVID-19 patients. The points reflect the mean expression level of the transcripts in RPKM.

To gather a relatively unbiased, transcriptome-wide view of the immune response to SARS-CoV2, Tempus-preserved whole blood RNA was subjected to RNAseq. Prior microarray-based profiling in the large MOSIAC trial identified a progression from interferon-induced to neutrophil-associated transcriptome changes in Tempus-preserved whole blood of patients with severe influenza [15]. For the current RNAseq analysis (n = 24), a subset of normal controls (n = 4) was combined with Incidental COVID patients (n = 3), and compared with combined Moderate and Critical COVID cases (n = 17). The results indicate that of the 44,783 total transcripts quantified at the gene level, 758 DEGs were identified between CON and COVIDs (S2 Table). Of these 758 DEGs, 603 transcripts were decreased in the COVID cases, while 155 transcripts were increased (4:1 decreased/increased ratio). Representative DEGs with their associated fold-changes are shown in Fig 1 (Panel A). The COVID-19 patients showed striking increases of >20-fold in known neutrophil-related transcripts such as Defensin alpha1 (DEFA1), matrix metallopeptidase 8 (MMP8), and myeloperoxidase (MPO). At least 67 of the 603 decreased transcripts were related to isoforms of the T-cell receptor. Also decreased were CD4, GATA3, ZAP70, TIGIT, IKZF2, ICOS and CD3D, intimately related to T cell function. The decreases in T-cell related transcripts are less striking in magnitude (3–5 fold) than the increased transcripts (20–25 fold), but T-cells (~5% of WBC) are a smaller subset of cells relative to neutrophils (>40% of WBC).

Gene ontology analysis indicated that the increased transcripts were most strongly associated with the innate immune response, with numerous additional associations to the immune response, especially defense against microorganisms (S2B Table). Transcripts decreased by COVID-19 were mapped to transcription and translation, with prominent associations to the adaptive immune response, T cells and TOR signalling (S2C Table). Pathway analysis indicated strong overlap with precurated pathways relevant to T helper cell differentiation and signaling, Th17 activation, T cell apoptosis and exhaustion (Fig 1, Panel B). Careful manual curation indicated that among the 155 increased transcripts, many were strongly associated with neutrophils or B-cells, while many of the 603 decreased transcripts were strongly specific to T-cells or NK cells.

### COVID-19 markedly alters cell-type-specific transcripts

To determine whether this cell-specific effect was due to an interpretation bias, the transcriptomes were compared to cell-type-specific transcriptomes from the Human Blood Atlas. The Blood Atlas’ data has been previously used to demonstrate enrichment in specific blood cell types in RNAseq samples [14], and was able to detect changes in T-cell abundance in coronary artery disease [16]. Average levels of the cell-type-specific transcripts are shown for control/incidental and critical patients (Fig 1, Panel C). Again, there is a relative increase in neutrophil- and B-cell-related transcripts and a decrease in T-cell related transcripts. Notably, the average increase in pre-curated neutrophil-specific transcripts (~3-fold, Panel C) is far less than the increase of some of the neutrophil-related DEGs (~25-fold, Panel A), suggesting that increased neutrophil counts alone do not fully explain the increased transcript levels. Rather, a plausible explanation is that transcript levels change during neutrophil “activation”, a broad term for the complex processes that lead to NETosis (neutrophil extracellular trap formation) and autolysis in response to various pathogen-related or environment-related stimuli.

Thus, the RNAseq provides very specific RNA-level evidence for the NLR imbalance that has been reported previously using more conventional cell counting methods. However, it is also possible that contributions of other cell types to these transcripts are relevant.

### Biomarker identification for COVID-19 syndrome

Among the transcripts significantly increased by COVID-19 were ALPL, DEFA1, DEFA3, and MPO, which we had previously identified in non-COVID infections, such as pneumonia and acute appendicitis. Also increased by COVID was the transcript for cathepsin G, a well-known neutrophil elastase with activity against viruses and bacteria. Cathepsin G (CTSG) was elevated 13-fold in COVIDs over controls (p<10^−5^). Neutrophils use 2 other elastases, neutrophil elastase (ELANE) and proteinase 3 (PR3, PRTN3), but their RNA levels were only detectable in some COVID patients. By comparison to several other studies from our lab and others, the magnitude of the increases in the neutrophil-related transcripts is quite strong, ranging from >3-fold for ALPL to >30-fold for MMP8, with substantial changes in the neutrophil defensins (20-25-fold) and myeloperoxidase (16-fold).

### RNAseq identifies biomarkers of COVID-19 severity

#### Severity defined by SARS-CoV2 seropositivity

The prior analysis compared controls to COVID-19 cases defined by clinical measures and SARS-CoV2 positivity in nasal swabs. However, the clinical severity of COVID-19 is affected by many factors that are independent of viral infectivity, especially pre-existing, comorbid conditions. Thus, to partially isolate the SARS-CoV2 contribution to disease severity, the controls were excluded, and the 20 COVID-19 patients were grouped according to seropositivity for SARS-CoV2 RNA in stabilized whole blood. Using a similar fold-change/p-value (>2-fold, <0.005) filtering of seropositive (n = 10) vs seronegative (n = 10) cases, 38 increased transcripts and 68 decreased transcripts were identified (S3A Table). A manually curated list of selected transcripts with useful annotations is shown in Table 2, and reinforces the conclusion that increased transcripts tend to be neutrophil/innate immunity related, while decreased transcripts are related to adaptive immunity, especially T cells.

**Table 2:** Selected transcripts differentially expressed in SARS-COV2 seropositive vs seronegative cases.

GeneSym	Description		SARS(-)		SARS(+)	FOLD	Putative Function in COVID-19
**Transcripts INCREASED in RNAemia (38 total)**			N = 10		N = 10	**CHANGE**	
ASPH	aspartate beta-hydroxylase		12.09		31.42	2.60	induces epitope-specific T cell responses
C5orf30	chromosome 5 open reading frame 30		3.79		14.69	3.88	autoimmune susceptibility, mphage resolution of inflammation
CDK5R1	cyclin-dependent kinase 5, regulatory subunit 1 (p35)		2.69		6.47	2.41	TGF-B sensitizes TRPV1 thru CDK5 signaling
CLEC4E	C-type lectin domain family 4 member E		73.86		167.84	2.27	damage associated molecular pattern DAMP
DAAM2	dishevelled associated activator of morphogenesis 2		0.80		7.54	9.41	formin protein, hypoxia, DAAM1 increased in SARS-ferret model
DGKH	diacylglycerol kinase eta		1.56		3.53	2.26	GWAS link to neutrophil abundance
ERLIN1	ER lipid raft associated 1		5.75		11.65	2.03	upregulated in sepsis, neutrophils
FKBP5	FK506 binding protein 5		27.28		76.85	2.82	regulates Influenza A infection with RIG-I
FLT3	fms related tyrosine kinase 3		3.38		12.21	3.62	related to COVID anticoagulation
GPR160	G protein-coupled receptor 160		7.23		15.67	2.17	regulates mycobacteria entry into macrophages
HMGB2	high mobility group box 2		102.94		223.43	2.17	VdJ recombination, mediates innate immunity to viral infection
INHBB	inhibin beta B		0.10		0.90	9.01	activin B, TGF-B axis
JDP2	Jun dimerization protein 2		8.09		17.90	2.21	GWAS to scrub typhus susceptibility, neutrophil
KCNE1	potassium voltage-gated channel subfamily E 1		1.99		4.96	2.49	novel target for immunomodulation in leukocytes
LINC00659	long intergenic non-protein coding RNA 659		0.10		1.36	13.56	high altitude thrombosis by spongeing mir-143, -15
OLAH	oleoyl-ACP hydrolase		2.64		21.57	8.16	increased in Influenza infection
PHC2	polyhomeotic homolog 2		45.55		97.97	2.15	detected in immune cells of RA
POLQ	polymerase (DNA) theta		0.73		1.82	2.50	somatic hypermutation of Ig genes
SAP30	Sin3A-associated protein		4.66		13.96	3.00	interacts with NS protein of Rift Valley Fever virus
SCRG1	stimulator of chondrogenesis 1		0.20		1.36	6.67	decreases tristetraprolin, autophagy related
SLC5A9	solute carrier family 5 member 9		0.10		0.97	9.72	SGLT4, mannose and fructose transporter
STS	steroid sulfatase (microsomal), isozyme S		1.99		4.14	2.08	interferon-gamma induced
TPST1	tyrosylprotein sulfotransferase 1		9.16		30.04	3.28	macrophage innate immune stimulated by TLR ligands
TSC22D3	TSC22 domain family member 3		108.24		263.41	2.43	related to T cell dysfunction
VSIG4	V-set and immunoglobulin domain containing 4		5.28		18.11	3.43	VSIG+ Macs suppress T cell proliferation, suppresses TLR4 path
ZNF608	zinc finger protein 608		2.43		15.92	6.56	increased in SARS-CoV2 Ferret model, B-cell
**Transcripts DECREASED in RNAemia (68 total)**							
A2M	alpha-2-macroglobulin		4.19		0.93	4.52	TGF-B scavenger
ACSM3	acyl-CoA synthetase medium-chain family member 3		2.56		1.04	2.46	blood pressure control
ANK3	ankyrin 3		2.12		0.75	2.84	T reg cells
CCR3	Receptor for a C-C type chemokine.		6.01		0.60	10.08	Th2 associated chemokine receptor
CD244	CD244 molecule		9.54		3.67	2.60	CD8 T and NK cytotoxicity
CNR2	cannabinoid receptor 2		1.88		0.31	6.14	macrophage related
CYSLTR2	Receptor for cysteinyl leukotrienes.		2.98		0.25	11.81	Leukotriene receptor
DZIP3	DAZ interacting zinc finger protein 3		3.00		1.26	2.38	induced by RSV infection
EPHA4	EPH receptor A4		2.42		0.63	3.85	activation of EPHA4 on CD4+CD45RO+ memory cells
FASLG	Fas ligand transcript variant 1		3.10		0.50	6.15	major regulator of apoptosis
GNLY	granulysin		75.96		24.73	3.07	stimulated CD8+ T cells
IL11RA	interleukin 11 receptor, alpha		3.79		1.76	2.15	T cell
IL5RA	interleukin 5 receptor subunit alpha		5.51		0.22	25.28	overexpressed in asthma, dermatitis, rhinitis
INPP4B	inositol polyphosphate-4-phosphatase type II B		7.48		2.97	2.52	corrlelates to testosterone and immune response in COVID19
LINC00612	long intergenic non-protein coding RNA 612		1.78		0.21	8.40	regulates Notch1 apoptosis, inflammation, and ox stress
LINC00861	long intergenic non-protein coding RNA 861		11.24		4.27	2.63	implicated in sepsis
LTBP4	latent transforming growth factor beta binding 4		2.11		0.67	3.15	TGF-B pathway critical for T reg differentiation
MAF	v-maf oncogene homolog		5.06		1.85	2.73	antiviral defense against Hep B virus
MIR151B	microRNA 151b		3.86		0.68	5.66	discriminates T from B cell lymphoma
MYBL1	MYB proto-oncogene like 1		4.81		1.35	3.57	T cell dependent B cell response
PZP	pregnancy-zone protein		1.69		0.10	16.92	biomarker of disease recovery COVID19
RNF182	ring finger protein 182		24.02		3.44	6.99	inhibits TLR-triggered cytokine production
RORA	RAR related orphan receptor A		7.71		3.12	2.47	T cell differentiation
TNFRSF25	tumor necrosis factor receptor superfamily, 25		5.01		1.86	2.69	aka DR3, costimulatory for T reg
TRAF1	TNF receptor associated factor 1		6.45		2.40	2.69	T and B cell
TR***	T Cell Receptor Associated (18 transcripts)		10.65		3.96	2.69	T cell receptor
UBASH3A	ubiquitin associated and SH3 domain containing A		5.40		1.69	3.19	T cell activation, negative regulator of activation
YBX1	Y-box binding protein 1		322.09		157.79	2.04	antibacterial response

### Severity defined by vasopressor use

**Fig 2:**
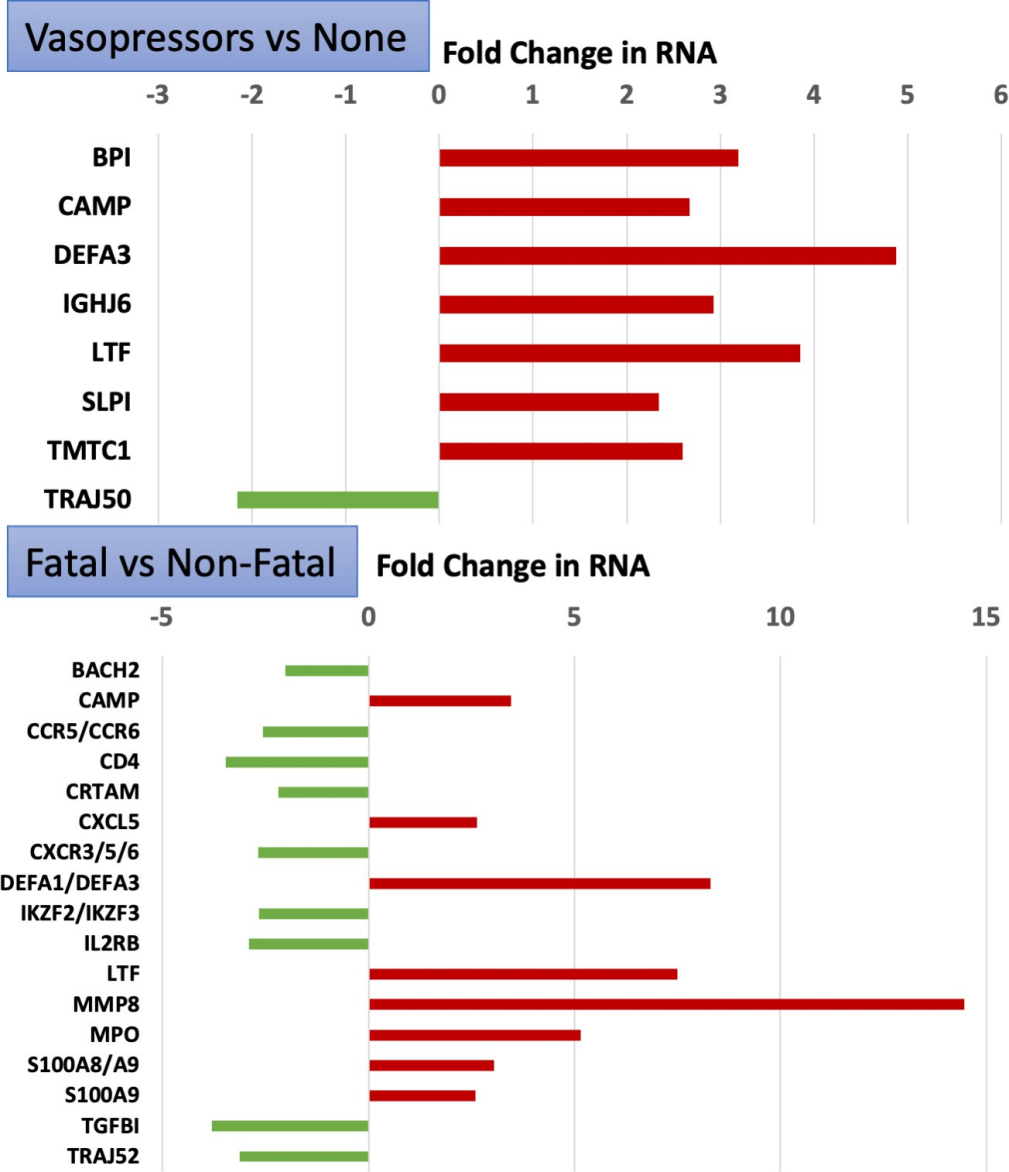
RNAseq of COVID-19 patients stratified by vasopressor use or fatality. The RNAseq data shown in Fig 1 was reanalyzed according to the severity of the COVID-19 using vasopressor use (upper panel) or fatality (lower panel) as the group criteria. DEGs were identified and examples of known transcripts are graphed with the fold-change increase (red bars) or decrease (green bars) of the patients relative to controls. Transcript gene symbols are shown on the left.

ICU patients with severe COVID-19 infections can become hypotensive, and thus are often given vasopressors, such as epinephrine and/or angiotensin II. Using vasopressor administration as an indicator of COVID-19 severity, the 24 critical patients were subdivided into Vasopressor (n = 9) and No-Vasopressor (n = 15) groups, and then reanalyzed for DEG at 2-fold change and p<0.05 uncorrected p-value. The results identified 33 DEG at the gene level, of which 11 were increased and 22 were decreased (S4 Table). The results echoed the overall results in the respect that COVID-19 patients on vasopressors had increased expression of neutrophil-like markers (BPI, CAMP, DEFA3) and decreased expression of T-cell related markers such as T-cell receptor alpha joining 50 (TRAJ50) (Fig 2).

#### Severity defined by survival

The critical COVID-19 patients were alternatively subdivided according to their survival, which identified 476 DEGs (S5 Table) and a similar trend was observed (Fig 2, lower panel). Patients that had a fatal outcome in the ICU (n = 5), when compared to survivors (n = 19), had drastically higher levels of RNAs for the neutrophil-related transcripts such as MMP8 (14-fold), MPO (5-fold) and DEFA1 or DEFA3 (8-fold). Conversely, the fatal COVID-19 patients exhibited 2–4 fold lower levels of T cell-related transcripts such as CD4, CXCR3, CXCR5, CXCR6, IKZF2/3, and T cell receptor alpha joining 52 (TRAJ52).

Notably, the patients that succumbed to COVID-19 displayed 2-fold lower levels of the CXCR6 transcript (Fig 2, lower panel), a memory T follicular helper (Tfh) marker, which was recently associated with COVID-19 severity by an expression quantitative trait loci (eQTL) analysis [17]. Furthermore, the current RNAseq results confirm ~30% of the transcripts identified by other labs using single cell RNAseq of PBMC of 6 mild vs 11 severe COVID-19 patients, much greater than expected by chance (see S6 Table for 22 near or exact matches, 18-fold enrichment, p = 6.3 x 10^−22^) [18].

Thus, a consistent trend emerges that the more severe the COVID-19, the more elevated are the neutrophil RNAs and more decreased are the T cell-related transcripts. This trend is consistent with previously published reports suggesting that the neutrophil/lymphocyte ratio (NLR) is one of the best correlates of COVID-19 disease severity [19–21].

### Blood RNA analysis by ddPCR confirms biomarkers in COVID-19 patients

The RNAseq analysis above, and prior blood transcriptome studies of patients with pneumonia or appendicitis, demonstrated elevated levels of five neutrophil-related RNAs: defensin a1 (DEFA1), alkaline phosphatase (ALPL), interleukin-8 receptor beta (IL8RB or CXCR2), myeloperoxidase (MPO) and resistin (RSTN). Their elevation was confirmed in a larger group of COVID patients using an independent method of RNA quantification.

**Fig 3:**
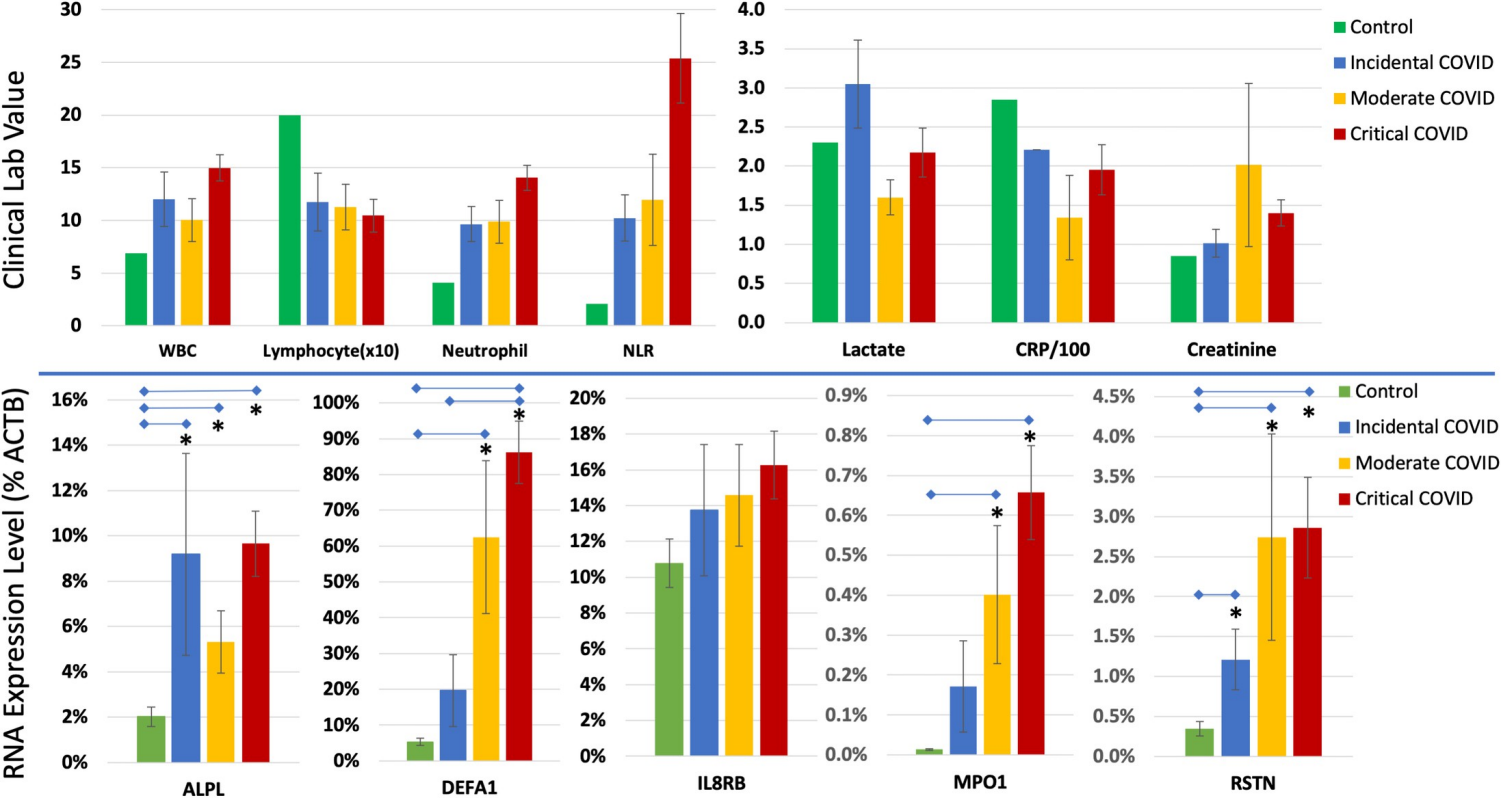
Clinical and RNA measures of COVID-19 severity. ***Upper panels*:** Patients testing positive for SARS-CoV2 by nasal swab PCR (n = 38) were divided into 3 groups of varying COVID-19 severity from Incidental (n = 7, asymptomatic and not the primary reason for admission), Moderate (n = 7, symptomatic but not on vasopressors or intubated), and Critical (n = 24, either vasopressors, intubated, or fatal). Reference levels (Control) are derived from published studies of normal controls. Bars reflect the white blood count (WBC), lymphocyte count (x10), neutrophil count, neutrophil/lymphocyte ratio (NLR), blood lactate, C-reactive protein (CRP), and creatinine for each group (mean ± s.e.m.). ***Lower panels*:** The same groups were used to calculate blood RNA levels for 5 transcripts, alkaline phosphatase (ALPL), defensin A1 (DEFA1), interleukin 8 receptor beta (IL8RB), myeloperoxidase 1 (MPO1), and resistin (RSTN), as a percentage of the actin B1 (ACTB) transcript used as the reference (i.e., ALPL in Incidental COVIDS is 9% of ACTB1 levels in copies per 20 μl of RNA in blood by ddPCR). Control subjects are unaffected normal subjects (n = 7) tested by the same process. (* = p < 0.05).

In the larger set of 38 patients from the ICU, the RNA biomarkers were quantified using droplet digital PCR (ddPCR) with gene-specific primers for cDNA synthesis. The patients were stratified by COVID symptom severity into incidental, moderate and critical COVID groups similar to the RNAseq study. Using conventional WBC and differential counts, there was a clear trend toward elevated WBC and neutrophil counts, decreased lymphocyte counts, and increased neutrophil/lymphocyte ratios (NLR), but these measures failed to differentiate between the severity groups with statistical significance (Fig 3). Likewise, lactate, CRP, and creatinine were not statistically different between groups. The RNA measures, however, showed striking and statistically significant differences between groups, with DEFA1, in particular, showing stepwise increases from 6.37% of actin B (%ACTB) in normal controls, to 19.7% in Incidental COVIDs, 62.5% in Moderate COVIDs, and 86.2% in Critical COVIDs (Fig 3, lower left panel). ALPL and IL8RB RNA levels, which we have shown are related to biofilm-type infections, such as appendicitis, also showed detectable increases. While expressed at overall lower absolute values than DEFA1, myeloperoxidase (MPO) and resistin (RSTN) RNAs showed strong and significant changes in the COVID-19 patients (Fig 3, lower right panels).

**Fig 4:**
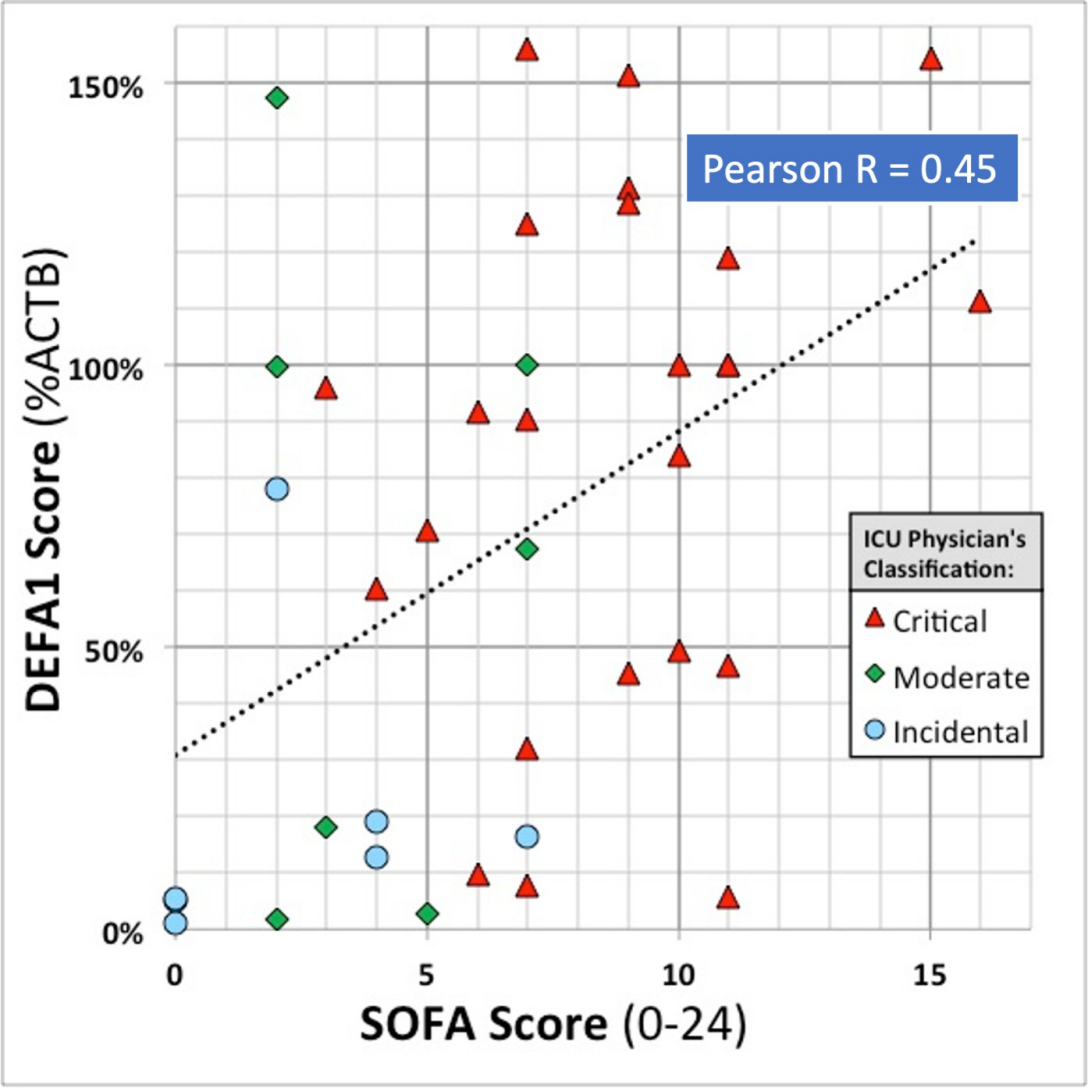
Whole blood DEFA1 RNA vs SOFA score of COVID-19 severity. Blood RNA from COVID-19 patients in the ICU (n = 38) was quantified for DEFA1 mRNA level by ddPCR and expressed as a percent of the ACTB mRNA level in the fixed RNA sample amount per patient (200 ng) thus minimizing changes in the abundance of neutrophils in blood between patients. The DEFA1 level (Y axis, as %ACTB) is plotted against the SOFA score determined clinically in the ICU according to the number and of degree of organ distress in the patient. Each point reflects a patient, colored according to their clinical classification. Dashed line indicates a linear fit to the data. R = Pearson’s correlation between SOFA and DEFA1.

The DEFA1 score in whole blood was modestly correlated with the Sequential Organ Failure Assessment (SOFA) score as an indicator of COVID-19 severity (Pearson R = 0.45, Fig 4). The SOFA score estimates the severity and number of organ systems involved in the disease. This analysis identified 3 patients with low to medium SOFA scores (6–11 on a scale of 24) that had apparently normal DEFA1 scores (<10%) that were classified as ‘Critical’. However, their viremia was at or below the threshold of detection of N1 or N2 spike protein transcripts by ddPCR, suggesting their illness may originate from non-SARS causes. Further, 2 of the 3 patients had elevated ‘biofilm’ biomarkers (APLP+IL8RB) suggesting they had non-SARS-CoV2 co-infections. The APACHE2 score was also positively, but slightly less correlated to DEFA1 score (Pearson R = 0.31, not shown).

### Sensitivity and specificity of DEFA1 RNA for detecting SARS seropositivity

Using whole blood RNA, SARS-CoV2 viremia (RNAemia) was measured by Bio-Rad ddPCR for 2 regions of the nucleocapsid protein in each of the whole blood RNA samples. Prior studies indicated that the presence of virus in plasma is associated with a worse prognosis [22, 23]. The DEFA1 score was stratified into 5 categories (1 = 0–5%, 2 = 6–10%, 3 = 11–20%, 4 = 21–50%, 5 = >51%) and compared to a binary classification of SARS-CoV2 using the specified thresholds for the ddPCR test. The results indicate that DEFA1 was 95.5% sensitive in detecting SARS-CoV2 infection, with 41.2% specificity, for overall 71.1% accuracy (ROC area = 0.697). However, it is important to note that many of SARS-CoV2 negative patients were known to have had other infections that could have triggered DEFA1 activation, and thus, the 41.2% specificity is unrealistically low. Only 1 of 21 ‘true’ positives was missed.

### Whole blood vs. purified neutrophil levels of RNA biomarkers of infection

**Fig 5:**
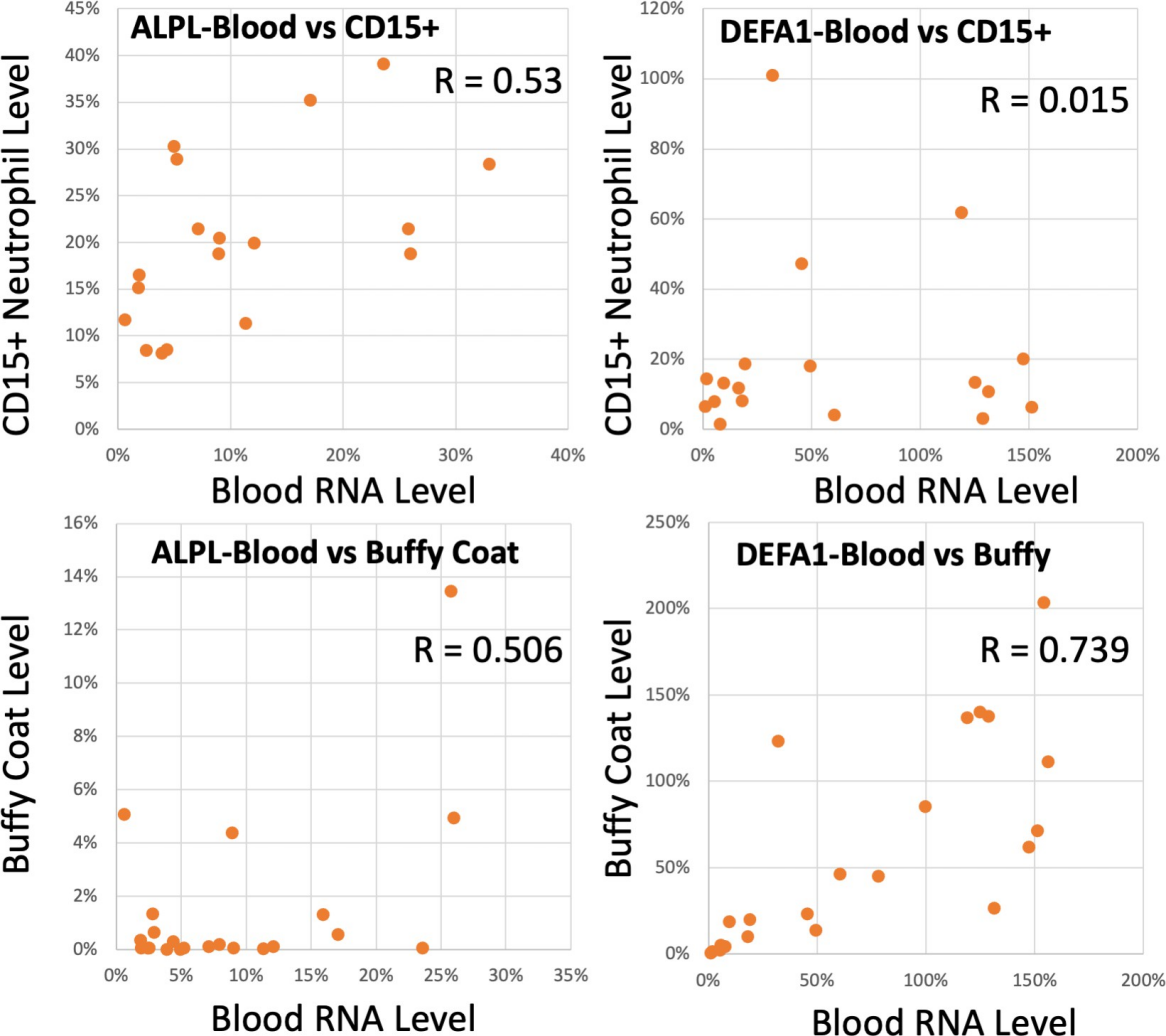
Whole blood RNA levels vs purified CD15+ neutrophil or buffy coat levels of immune activation markers. In a subset of COVID-19 patients, whole blood RNA was compared to RNA from purified CD15+ neutrophils (n = 16) or buffy coat (n = 22) from the same patients for 6 mRNA biomarkers using ddPCR quantitation. Each point represents a single patient quantified for ALPL (left panels) or DEFA1 (right panels) RNA in whole blood RNA vs purified CD15+ neutrophils (upper panels) or buffy coat RNA (lower panels). All RNA levels are expressed as % of ACTB in the same ddPCR measurement.

Because DEFA1, in particular, is considered a neutrophil defensin, it was hypothesized that the DEFA1 signal would be highly enriched in isolated CD15+ cells, including >70% of the whole blood’s neutrophils. Thus, in a subset of 23 patients, the total blood RNA, the CD15+ neutrophil RNA, and the buffy coat RNA were compared for levels of DEFA1, ALPL, IL8RB, MPO1, and RSTN (Fig 5). Surprisingly, the levels of these markers, especially DEFA1, was essentially uncorrelated between whole blood and CD15+ cells (Pearson R = 0.015, Fig 5, upper right panel). ALPL (R = 0.53) showed a more positive, but still modest correlation between the 2 sample types (upper left panel), while IL8RB (R = 0.04) was also uncorrelated between whole blood and purified CD15+ neutrophils (not shown). While the average absolute level of DEFA1 RNA was actually lower in CD15+ neutrophils than whole blood (20.4% vs. 68%), the ALPL (20.1% vs. 10.1%) and IL8RB levels (42.7% vs. 17.7%) were enriched in the CD15+ cells, consistent with the known localization of those markers to neutrophils.

Surprisingly, the buffy coat RNA demonstrated the best correlation to whole blood RNA (Fig 5, lower panels). Comparison of CD15+ neutrophil RNA to buffy coat RNA from the same subjects showed a relatively strong correlation between DEFA1 levels (r = 0.74), ALPL (0.51), and IL8RB (0.41), suggesting the RNA infection signals transcend the neutrophil subset. Buffy coat RNA exhibited a DEFA1 mRNA level about as strong as the whole blood of the same patients (58.9% vs 68% in whole blood). The ALPL (1.52%) and IL8RB (2.60%) signals were strikingly lower in buffy coat compared to the CD15+ neutrophils (ALPL = 20.1%, IL8RB = 42.7%) or whole blood RNA (ALPL = 10.1%, IL8RB = 17.7%), consistent with the belief that they are relatively ‘neutrophil-specific’ markers.

There are several potential explanations for the strong correlation of the buffy coat RNA levels compared to neutrophil levels of these RNA biomarkers. First, some neutrophils, especially immature ‘band’ neutrophils, segregate into the buffy coat, as opposed to the heavier neutrophils that sediment with RBC [24]. Secondly, the buffy coat contains other cells such as monocytes which could express these RNAs, despite being considered ‘neutrophil-specific’ transcripts. Third, the CD15+ cells undergo a relatively lengthy isolation process of about 1 hour in which they are viable and could be inducing or degrading particular RNAs. By comparison, the buffy coat isolation is relatively fast, and might retain the whole blood signal better than purified PMNs.

### CD15+ cells from COVID-19 patients show increased nuclear chromatin staining

**Fig 6:**
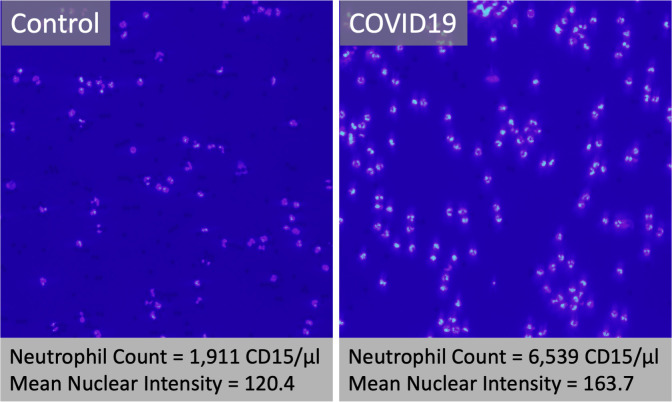
Fluorescent DNA stain of purified CD15+ neutrophils from control or COVID-19 patients. Purified CD15+ neutrophils were purified from whole blood of Control (left panel) or COVID-19 patients (right panel) using antibody-coated paramagnetic beads. Washed cells were bound magnetically to a glass slide, briefly fixed, and stained with DAPI, which fluoresces only when intercalated on double strand DNA. The neutrophil count and mean nuclear intensity were quantified for digital images at identical exposures using image analysis (ImageJ, NIH). Reported values reflect mean of 3 random 20X fields per subject.

Based on the elevated neutrophil RNA levels of DEFA1, it was hypothesized that CD15+ neutrophils from COVID-19 patients might show visible signs of cellular activation. Affinity purified CD15+ neutrophils were isolated from a subset of patients with or without COVID-19 and then magnetically adhered to glass slides for histological analysis. Vital (Hoechst 33342) and fixed staining (DAPI) of the nuclear chromatin were performed on CD15+ neutrophils from COVID-19 patients. Patients from the groups with more severe symptoms showed increased neutrophil numbers and increased intensity of nuclear chromatin staining, suggesting increased accessibility of the chromatin to uptake the stain (Fig 6). To our knowledge, this is the first report of heightened nuclear fluorescence (DAPI^bright^) staining of neutrophils in relation to human infection.

### CD15+ cells from COVID-19 patients demonstrate elevated neutrophil elastase activity

**Fig 7:**
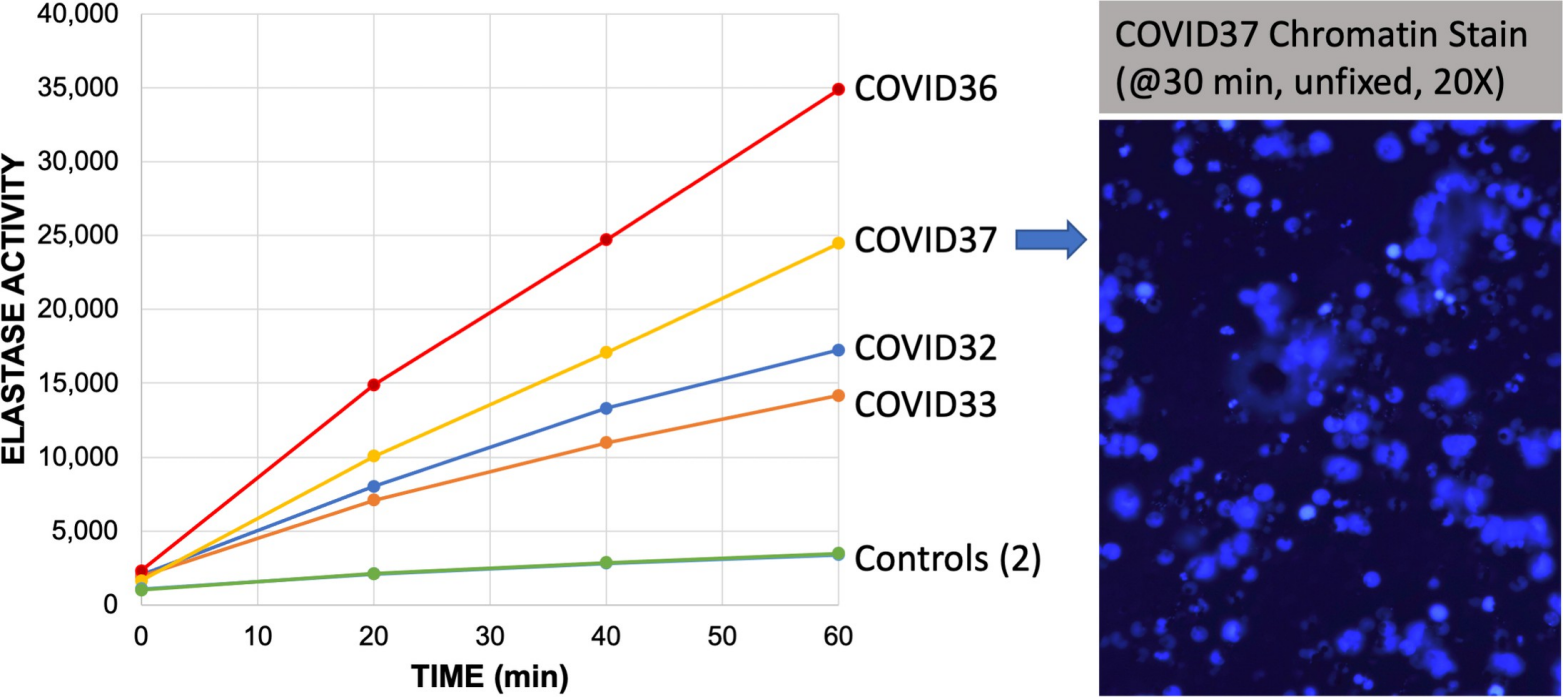
Neutrophil elastase activity of purified CD15+ neutrophils from control or COVID-19 patients. CD15+ neutrophils were purified from whole blood of patients or controls using antibody coated magnetic beads, which were then counted using a hemocytometer. The CD15+ neutrophils from the equivalent of 50 μl of blood (250K-600K cells) were then freeze-thawed (5X) and assayed for elastase activity using a fluorometric substrate measured over time for 4 COVID-19 patients (i.e. COVID36, COVID37, etc.). Each point is a single measure of fluorescence at the specified times after adding substrate. Controls (n = 2) are essentially superimposed. In the right panel, parallel samples of CD15+ neutrophils were magnetically adhered to a glass slide in HBSS and allowed to attach for 30 min prior to staining with DAPI to image the DNA without fixation. Photomicrograph is stimulated at 375 nm and emission captured at 415–460 nm with a 20X objective.

Nuclear chromatin decompaction is partially attributed to translocation of neutrophil elastase from granules to the nucleus, and is one of the early steps in the assembly and release of neutrophil extracellular traps (NETs). Elastolytic enzymes, such as neutrophil elastase (NE) and cathepsin G, cleave the histones that maintain chromatin packing. Prior studies have shown that biomarkers of NETosis, such as cell free DNA (cfDNA), extracellular histone H3, and NE are elevated in COVID19 patients and correlate with the severity of disease [25]. Thus, the hypothesis that NE activity was increased by COVID-19 infection was tested *ex vivo*. NE activity was measured in purified, lysed CD15+ neutrophils using a kinetic assay that measured the cleavage of a fluorescent peptide substrate over time (Fig 7). The number of CD15+ neutrophils assayed was equivalent to the number of neutrophils in a fingerstick volume (50 μl) of the patient’s whole blood. Not surprisingly, the number of CD15+ cells per 50 μl was 5.2-fold higher in COVID-19 patients (n = 10, avg. = 7.05 x 10^5^ cells) than in in controls (n = 4, avg. = 1.35 x 10^5^ cells; p = 0.25). The elastase activity in the COVID-19 patients (4 shown) was markedly higher than 2 uninfected control subjects (Fig 7, left panel). In parallel to the NE assay, the same COVID patients’ CD15+ cells were chromatin-stained at 30 minutes, and they demonstrated striking adhesion, spreading, and NETosis, as shown for one patient (right panel).

**Fig 8:**
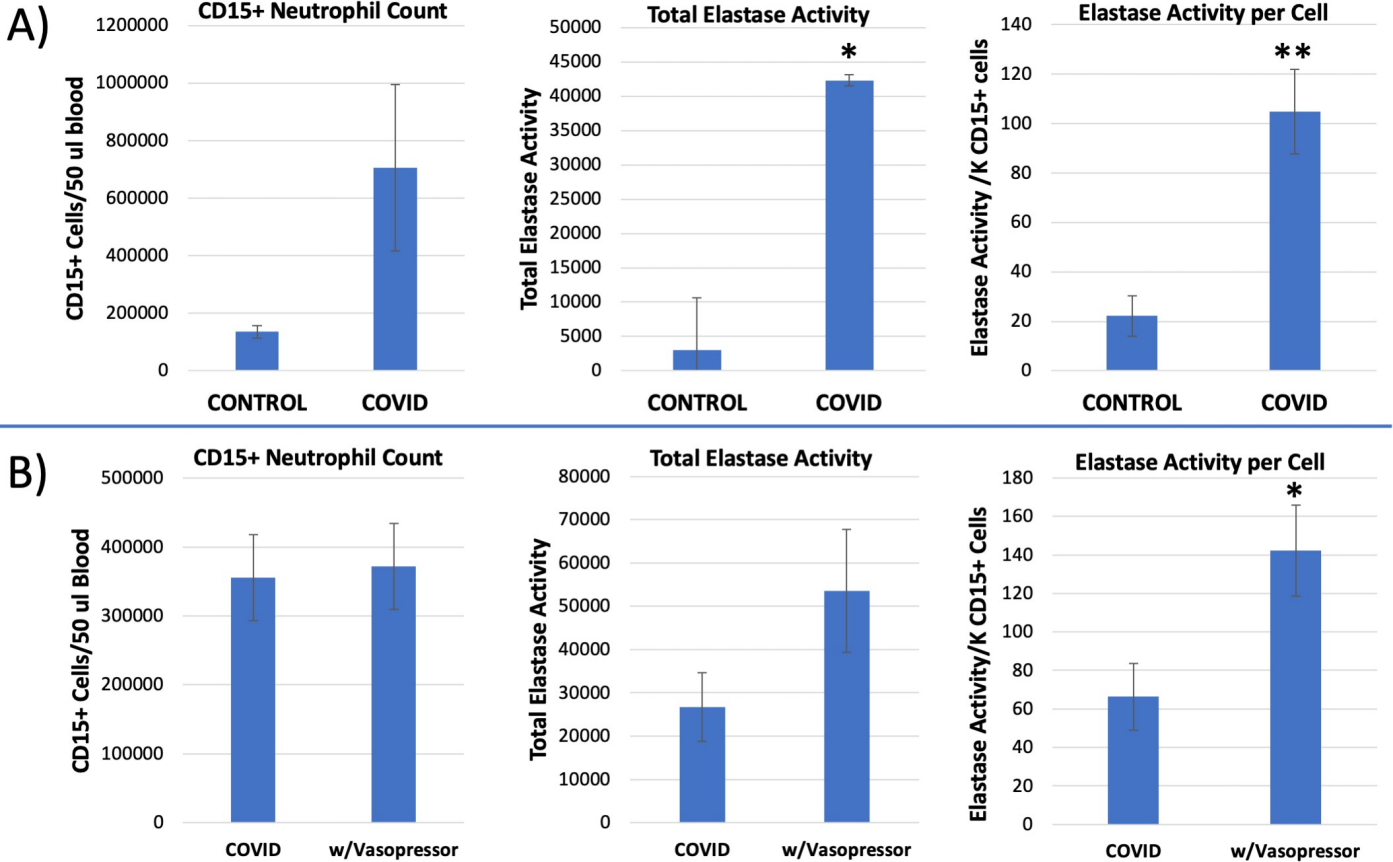
Neutrophil elastase activity of purified CD15+ neutrophils from control or COVID-19 patients. **Panel A)** Using the kinetic elastase assay shown in Fig 7, COVID-19 patient’s purified CD15+ neutrophils (n = 10) were compared to control subjects (n = 4). The left panel reports the total cell count of CD15+ neutrophils from 50 μl of blood (mean ± s.e.m.). The total elastase activity of those cells was measured in arbitrary fluorescence units per 2 hour incubation (middle panel, mean ± s.e.m.). The total elastase activity per patient was divided by the CD15+ cell count for that patient to yield the elastase activity per cell (right panel, mean ± s.e.m.). **Panel B)** Patients that were PCR+ for SARS-CoV2, were regrouped according to whether they received vasopressor support (n = 4) or did not receive vasopressors (n = 8), (* = p<0.05, ** = p<0.01).

As shown in Fig 8, total elastase activity in purified CD15+ cells was increased more than 13-fold in COVID-19 patients (n = 10) relative to Controls (n = 4, p = 0.008). Compensating for the increased numbers of neutrophils in COVID-19 patients, which was elevated about 3-fold in this larger group (Fig 8A), demonstrates that CD15+ neutrophils had a 4.7-fold increased neutrophil elastase activity per cell (p = 0.012). This is a surprising and potentially important finding because it was not previously known that the elastase activity per cell increased in human viral infections. Thus, the increased elastase observed in COVID-19 patients is due to both an increase in the number of CD15+ neutrophils, and an increase in the elastase activity produced by each cell.

The level of elastase activity in the purified CD15+ neutrophils appeared to be related to the severity of the infection, as shown in Fig 8B. Comparing COVID-19 patients that did not need vasopressor support (n = 8) with those that did need vasopressor support (n = 4) indicates that while the CD15+ cell count was not significantly different between groups (355K vs 372K, respectively), the neutrophil elastase activity per cell was increased >2-fold in the patients needing vasopressor support (p<0.05). By comparison, in this same smaller group of patients, neither the NLR (no pressor = 10.82 vs pressor = 6.57, n = 7 vs 3, ns) nor the lactate levels (no pressor = 2.25 vs pressor = 2.13, n = 4 vs 3, ns) were different between no pressors and pressor patients.

The clinical consequence of increased elastase activity in COVID-19 patients is not clear. While enhanced NE activity might help to clear extracellular SARS-CoV2, other studies indicate that human neutrophil elastase’s cleavage of the spike protein within an activation loop region may enhance fusion of the viral envelope with host cell membranes [26]. NE levels in plasma, and other NETosis markers, are elevated in COVID-19 cases and may have prognostic value toward severity [3, 11, 25]. However, NE activity may be more than a biomarker of neutrophil activation because early mutations in the SARS-CoV2 spike protein created elastase cleavage sites that seem to enhance viral spread [27, 28]. Overall, functional analysis suggests that the virus does not replicate well in neutrophils [29].

### Plasma elastase activity in COVID-19 patients

**Fig 9:**
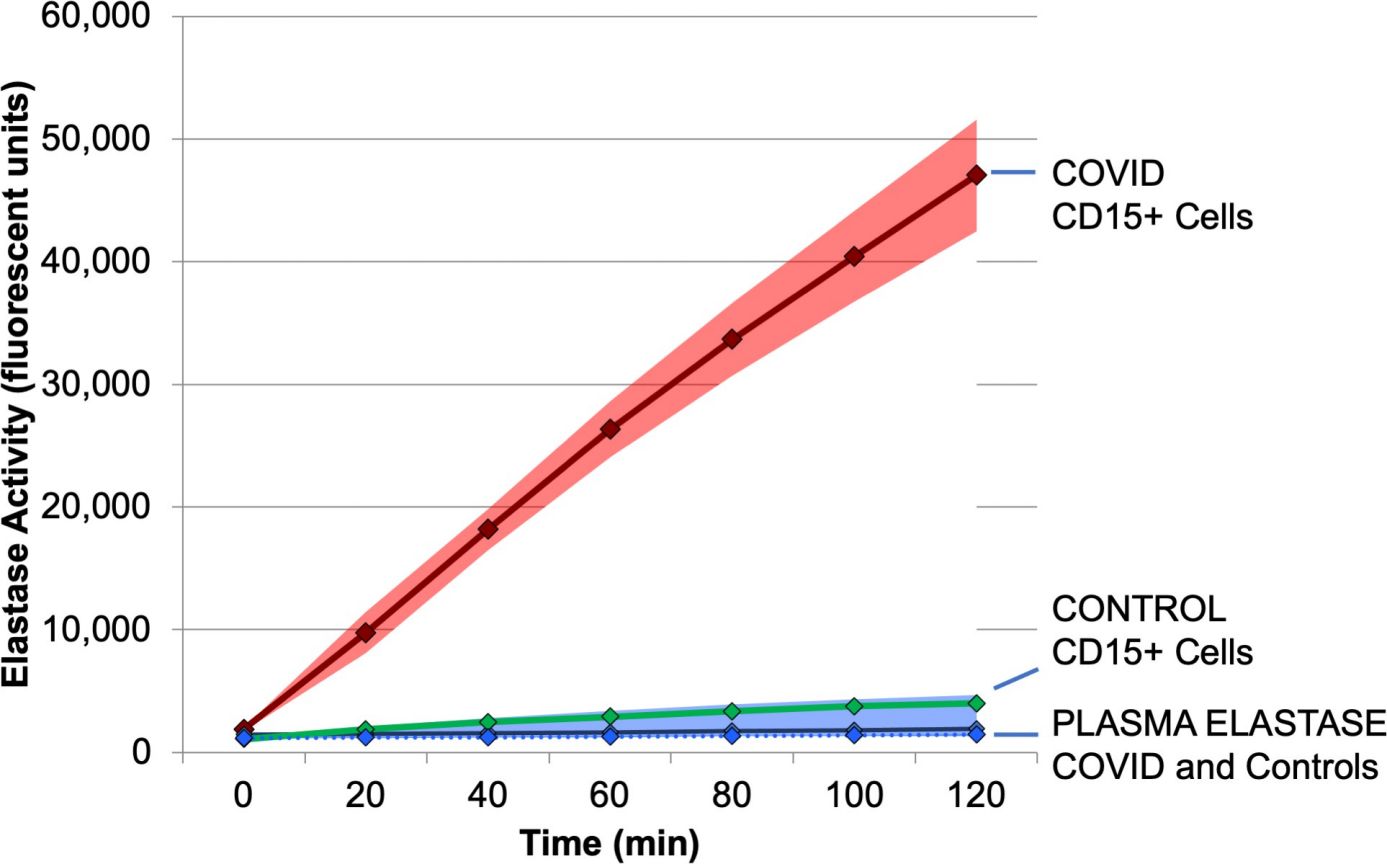
Neutrophil elastase activity of purified CD15+ neutrophils vs. autologous plasma from control or COVID-19 patients. Elastase activity, in relative fluorescence units (Y Axis) was measured in freeze-thaw lysates of purified CD15+ neutrophils from COVID-19 patients (n = 5, RED line) versus plasma (n = 5, BLUE solid line) from the same patients. Points reflect the mean fluorescence (Y axis) at each time point (X axis) with 95% confidence intervals in light red or blue shading. For reference, elastase active from CD15+ neutrophils (GREEN) or plasma (BLUE dashed) of normal controls (n = 3). Plasma elastase from COVID-19 and Controls are low and nearly superimposed.

Because the CD15+ neutrophils had elevated elastase activity in COVID-19 patients, it raised the possibility that circulating plasma levels of elastase might also be elevated, making a convenient biomarker of COVID-19 infection. Neutrophil elastase protein, measured antigenically in plasma, was increased in COVID-19 patients [11, 29]. However, in a subset of the current cohort, the elastase activity in their plasma did not correlate well with their status as control or COVID-19 patients (Fig 9). The plasma (50 μl) was used in equivalent volume to the CD15+ cells, and was treated identically with respect to lysis method (freeze-thaw), substrate concentration, and time. Overall, from analysis of 5 COVID patients, with known high elastase activity in CD15+ cells, versus 3 control patients, elastase activity in plasma was detected at only 1% of the level detected in the plasma (Fig 9).

### Limitations of the current study

Because of the difficulty in consenting and studying severely ill COVID-19 patients in the midst of the pandemic, the sample size for the current study is modest. As our studies began to identify NE as a potential biomarker and point-of-care assay, the vaccines became available and were prevalent in the DC area, fortunately reducing the number of COVID-19 cases seen at our hospital. A surprising limitation was the variable certainty with which the clinical syndrome could be attributed to SARS-CoV2 infection. Some patients that were asymptomatic and were only included because of positive SARS-CoV2 nasal swab as a part of routine surveillance, turned out to have very high blood titer of virus. Conversely, some very clinically ill patients did not have high viral RNAemia, which could indicate that the virus was highly localized in the lungs, for instance, or that the RNAemia had cleared, but secondary, opportunistic infections had prevailed.

## Conclusions

COVID-19 syndrome triggers potent changes in the blood transcriptome as measured by RNAseq, and confirmed on selected targets by ddPCR. The severity of COVID-19 is associated with significant changes in the activation of neutrophil-related transcripts, such as DEFA1, and decreased levels of T cell-related transcripts, such as the T cell receptor. Seropositivity for SARS-CoV2, as measured by ddPCR of blood RNA, was detected at 95.5% sensitivity by an elevated DEFA1 RNA in blood. Purification of CD15+ neutrophils from COVID-19 patients did not markedly enrich for these ‘neutrophil-specific’ transcripts, suggesting other cell types may contribute to their elevation in the whole blood RNA of infected persons. Isolated CD15+ neutrophils from COVID-19 patients showed striking increases in nuclear DNA staining with DAPI, consistent with the early steps of chromatin remodeling in NETosis. The COVID-19 neutrophils likewise exhibited >5-fold elevated neutrophil elastase activity on a per-cell basis, consistent with the elevated transcript levels of cathepsin G (CTSG) and DEFA1, and the de-compacted, DAPI^bright^ chromatin. Circulating plasma levels of elastase activity in COVID-19 patients was very low (~1%) compared to CD15+ neutrophil elastase activity of the same patients. Neutrophil elastase has the potential to be useful as a rapid point-of-care assay for the detection of neutrophil activation.

## Supporting information

S1 Table Additional demographic parameters of subjects with statistical analysis. (XLSX)

S2 Table All differentially expressed genes (DEGs) between COVID and Controls. (XLSX)

S3 Table Complete RNAemia DEGs summarized in Table 2. (XLSX)

S4 Table DEGs related to vasopressor status. (XLSX)

S5 Table DEGs related to fatality from COVID19. (XLSX)

S6 Table DEGs matching prior report by Notaborollo et al. (XLSX)

## Abbreviations

CBC: complete blood count
ddPCR: droplet digital PCR
DEGs: differentially expressed genes
RIN: RNA integrity number
RNAseq: RNA sequencing
rRNA: ribosomal RNA
RPKM: reads per kilobase of exon per million mapped total reads

## References

[1] Qian Z, Lu S, Luo X, Chen Y, Liu L. Mortality and Clinical Interventions in Critically ill Patient With Coronavirus Disease 2019: A Systematic Review and Meta-Analysis. Front Med (Lausanne). 2021;8:635560. Epub 2021/08/10. doi: 10.3389/fmed.2021.635560 ; PubMed Central PMCID: PMC8342953. 34368175 PMC8342953

[2] Chawla LS, Toma I, Davison D, Vaziri K, Lee J, Lucas R, et al. Acute appendicitis: transcript profiling of blood identifies promising biomarkers and potential underlying processes. BMC Med Genomics. 2016;9(1):40. doi: 10.1186/s12920-016-0200-y ; PubMed Central PMCID: PMC4946184. 27417541 PMC4946184

[3] Jackson HT, Mongodin EF, Davenport KP, Fraser CM, Sandler AD, Zeichner SL. Culture-independent evaluation of the appendix and rectum microbiomes in children with and without appendicitis. PloS one. 2014;9(4):e95414. doi: 10.1371/journal.pone.0095414 ; PubMed Central PMCID: PMC3997405. 24759879 PMC3997405

[4] Maibach MA, Allam A, Hilty MP, Perez Gonzalez NA, Buehler PK, Wendel Garcia PD, et al. How to Synchronize Longitudinal Patient Data With the Underlying Disease Progression: A Pilot Study Using the Biomarker CRP for Timing COVID-19. Front Med (Lausanne). 2021;8:607594. Epub 2021/07/27. doi: 10.3389/fmed.2021.607594 ; PubMed Central PMCID: PMC8295502. 34307391 PMC8295502

[5] Musuuza JS, Watson L, Parmasad V, Putman-Buehler N, Christensen L, Safdar N. Prevalence and outcomes of co-infection and superinfection with SARS-CoV-2 and other pathogens: A systematic review and meta-analysis. PloS one. 2021;16(5):e0251170. Epub 2021/05/07. doi: 10.1371/journal.pone.0251170 ; PubMed Central PMCID: PMC8101968. 33956882 PMC8101968

[6] Catano-Correa JC, Cardona-Arias JA, Porras Mancilla JP, Garcia MT. Bacterial superinfection in adults with COVID-19 hospitalized in two clinics in Medellin-Colombia, 2020. PloS one. 2021;16(7):e0254671. Epub 2021/07/14. doi: 10.1371/journal.pone.0254671 ; PubMed Central PMCID: PMC8277025. 34255801 PMC8277025

[7] Iwamura APD, Tavares da Silva MR, Hummelgen AL, Soeiro Pereira PV, Falcai A, Grumach AS, et al. Immunity and inflammatory biomarkers in COVID-19: A systematic review. Rev Med Virol. 2021;31(4):e2199. Epub 2021/07/15. doi: 10.1002/rmv.2199 . 34260778

[8] Man MA, Rajnoveanu RM, Motoc NS, Bondor CI, Chis AF, Lesan A, et al. Neutrophil-to-lymphocyte ratio, platelets-to-lymphocyte ratio, and eosinophils correlation with high-resolution computer tomography severity score in COVID-19 patients. PloS one. 2021;16(6):e0252599. Epub 2021/06/29. doi: 10.1371/journal.pone.0252599 . 34181675 PMC8238190

[9] Mehta P, Fajgenbaum DC. Is severe COVID-19 a cytokine storm syndrome: a hyperinflammatory debate. Curr Opin Rheumatol. 2021;33(5):419–30. Epub 2021/07/16. doi: 10.1097/BOR.0000000000000822 ; PubMed Central PMCID: PMC8373392. 34264880 PMC8373392

[10] Melo AKG, Milby KM, Caparroz A, Pinto A, Santos RRP, Rocha AP, et al. Biomarkers of cytokine storm as red flags for severe and fatal COVID-19 cases: A living systematic review and meta-analysis. PloS one. 2021;16(6):e0253894. Epub 2021/06/30. doi: 10.1371/journal.pone.0253894 ; PubMed Central PMCID: PMC8241122. 34185801 PMC8241122

[11] Ng H, Havervall S, Rosell A, Aguilera K, Parv K, von Meijenfeldt FA, et al. Circulating Markers of Neutrophil Extracellular Traps Are of Prognostic Value in Patients With COVID-19. Arterioscler Thromb Vasc Biol. 2021;41(2):988–94. Epub 2020/12/04. doi: 10.1161/ATVBAHA.120.315267 ; PubMed Central PMCID: PMC7837697. 33267662 PMC7837697

[12] Szturmowicz M, Demkow U. Neutrophil Extracellular Traps (NETs) in Severe SARS-CoV-2 Lung Disease. Int J Mol Sci. 2021;22(16). Epub 2021/08/28. doi: 10.3390/ijms22168854 . 34445556 PMC8396177

[13] Consortium M, Shi L, Reid LH, Jones WD, Shippy R, Warrington JA, et al. The MicroArray Quality Control (MAQC) project shows inter- and intraplatform reproducibility of gene expression measurements. Nat Biotechnol. 2006;24(9):1151–61. Epub 2006/09/12. doi: 10.1038/nbt1239 ; PubMed Central PMCID: PMC3272078. 16964229 PMC3272078

[14] Uhlen M, Karlsson MJ, Zhong W, Tebani A, Pou C, Mikes J, et al. A genome-wide transcriptomic analysis of protein-coding genes in human blood cells. Science. 2019;366(6472). Epub 2019/12/21. doi: 10.1126/science.aax9198 . 31857451

[15] Dunning J, Blankley S, Hoang LT, Cox M, Graham CM, James PL, et al. Progression of whole-blood transcriptional signatures from interferon-induced to neutrophil-associated patterns in severe influenza. Nat Immunol. 2018;19(6):625–35. Epub 2018/05/20. doi: 10.1038/s41590-018-0111-5 ; PubMed Central PMCID: PMC5985949. 29777224 PMC5985949

[16] McCaffrey TA, Toma I, Yang Z, Katz R, Reiner J, Mazhari R, et al. RNA sequencing of blood in coronary artery disease: involvement of regulatory T cell imbalance. BMC Med Genomics. 2021;14(1):216. Epub 2021/09/05. doi: 10.1186/s12920-021-01062-2 ; PubMed Central PMCID: PMC8414682. 34479557 PMC8414682

[17] Kasela S, Daniloski Z, Bollepalli S, Jordan TX, tenOever BR, Sanjana NE, et al. Integrative approach identifies SLC6A20 and CXCR6 as putative causal genes for the COVID-19 GWAS signal in the 3p21.31 locus. Genome Biol. 2021;22(1):242. Epub 2021/08/25. doi: 10.1186/s13059-021-02454-4 . 34425859 PMC8381345

[18] Notarbartolo S, Ranzani V, Bandera A, Gruarin P, Bevilacqua V, Putignano AR, et al. Integrated longitudinal immunophenotypic, transcriptional and repertoire analyses delineate immune responses in COVID-19 patients. Science Immunology. 2021;6. doi: 10.1126/sciimmunol.abg5021 34376481

[19] Urrechaga E, Mugertza G, Fernandez M, Espana PP, Aguirre U. Leukocyte differential and reactive lymphocyte counts from Sysmex XN analyzer in the evaluation of SARS-CoV-2 infection. Scand J Clin Lab Invest. 2021:1–7. Epub 2021/06/10. doi: 10.1080/00365513.2021.1929445 . 34106799

[20] Sayed AA, Allam AA, Sayed AI, Alraey MA, Joseph MV. The use of neutrophil-to-lymphocyte ratio (NLR) as a marker for COVID-19 infection in Saudi Arabia: A case-control retrospective multicenter study. Saudi Med J. 2021;42(4):370–6. Epub 2021/04/03. doi: 10.15537/smj.2021.42.4.20200818 . 33795491 PMC8128643

[21] Chen L, Yu J, He W, Chen L, Yuan G, Dong F, et al. Risk factors for death in 1859 subjects with COVID-19. Leukemia. 2020;34(8):2173–83. Epub 2020/06/18. doi: 10.1038/s41375-020-0911-0 ; PubMed Central PMCID: PMC7296516. 32546725 PMC7296516

[22] Pena KB, Riu F, Guma J, Guilarte C, Pique B, Hernandez A, et al. Study of the Plasma and Buffy Coat in Patients with SARS-CoV-2 Infection-A Preliminary Report. Pathogens. 2021;10(7). Epub 2021/07/03. doi: 10.3390/pathogens10070805 ; PubMed Central PMCID: PMC8308615. 34201967 PMC8308615

[23] Miki S, Sasaki H, Horiuchi H, Miyata N, Yoshimura Y, Miyazaki K, et al. On-admission SARS-CoV-2 RNAemia as a single potent predictive marker of critical condition development and mortality in COVID-19. PloS one. 2021;16(7):e0254640. Epub 2021/07/14. doi: 10.1371/journal.pone.0254640 ; PubMed Central PMCID: PMC8277033. 34255796 PMC8277033

[24] Teetson W, Cartwright C, Dreiling BJ, Steinberg MH. The leukocyte composition of peripheral blood buffy coat. Am J Clin Pathol. 1983;79(4):500–1. Epub 1983/04/01. doi: 10.1093/ajcp/79.4.500 6837515

[25] Huckriede J, Anderberg SB, Morales A, de Vries F, Hultstrom M, Bergqvist A, et al. Evolution of NETosis markers and DAMPs have prognostic value in critically ill COVID-19 patients. Sci Rep. 2021;11(1):15701. Epub 2021/08/05. doi: 10.1038/s41598-021-95209-x ; PubMed Central PMCID: PMC8333321. 34344929 PMC8333321

[26] Mustafa Z, Zhanapiya A, Kalbacher H, Burster T. Neutrophil Elastase and Proteinase 3 Cleavage Sites Are Adjacent to the Polybasic Sequence within the Proteolytic Sensitive Activation Loop of the SARS-CoV-2 Spike Protein. ACS Omega. 2021;6(10):7181–5. Epub 2021/03/23. doi: 10.1021/acsomega.1c00363 ; PubMed Central PMCID: PMC7970549. 33748632 PMC7970549

[27] Bhattacharyya C, Das C, Ghosh A, Singh AK, Mukherjee S, Majumder PP, et al. SARS-CoV-2 mutation 614G creates an elastase cleavage site enhancing its spread in high AAT-deficient regions. Infect Genet Evol. 2021;90:104760. Epub 2021/02/09. doi: 10.1016/j.meegid.2021.104760 ; PubMed Central PMCID: PMC7863758. 33556558 PMC7863758

[28] Pokhrel S, Kraemer BR, Lee L, Samardzic K, Mochly-Rosen D. Increased elastase sensitivity and decreased intramolecular interactions in the more transmissible 501Y.V1 and 501Y.V2 SARS-CoV-2 variants’ spike protein-an in silico analysis. PloS one. 2021;16(5):e0251426. Epub 2021/05/27. doi: 10.1371/journal.pone.0251426 ; PubMed Central PMCID: PMC8153447. 34038453 PMC8153447

[29] Metzemaekers M, Cambier S, Blanter M, Vandooren J, de Carvalho AC, Malengier-Devlies B, et al. Kinetics of peripheral blood neutrophils in severe coronavirus disease 2019. Clin Transl Immunology. 2021;10(4):e1271. Epub 2021/05/11. doi: 10.1002/cti2.1271 ; PubMed Central PMCID: PMC8082714. 33968405 PMC8082714

