# Supplementary material for: RNA Sequencing in COVID-19 patients identifies neutrophil activation biomarkers as a promising diagnostic platform for infections
Source: PLoS One. 2022 Jan 26;17(1):e0261679. doi: 10.1371/journal.pone.0261679 (PMC8791486; doi:10.1371/journal.pone.0261679)
Supplement: Attachment — Submitted filename: COVID PLOS Response V2.docx [file pone.0261679.s007.docx]

PONE-D-21-30161

RNA Sequencing in COVID-19 patients identifies neutrophil activation biomarkers as promising diagnostic platform for infections.

PLOS ONE

**Editor #1.** Please ensure that your manuscript meets PLOS ONE's style requirements, including those for file naming. The PLOS ONE style templates can be found at…

**Response:** We have checked the revised manuscript, especially file naming, against the style template. We made numerous formatting adjustments, and added line numbers.

**Editor #**2. Please provide additional details regarding participant consent. In the ethics statement in the Methods and online submission information, please ensure that you have specified what type you obtained (for instance, written or verbal, and if verbal, how it was documented and witnessed). If your study included minors, state whether you obtained consent from parents or guardians.

**Response:** This has been corrected in the revised manuscript.

**Editor #**3. We note that you have provided funding information within the Funding Section. Please remove any funding-related text from the manuscript and let us know how you would like to update your Funding Statement.

**Response:** We have removed the funding statement from the manuscript.

Please update the online Funding Statement to read as follows:

“The authors are grateful for the generous financial support of True Bearing Diagnostics, Inc., The St. Laurent Institute, and The Ulvi and Reykhan Kasimov Family. The authors are also grateful for the institutional support provided by the CTSI-CN Award Number UL1TR001876 from the NIH National Center for Advancing Translational Sciences, and the Core Instrument Grant for the Bio-Rad ddPCR S10 OD021622.”

**Editor #**4. Please state what role the funders took in the study.

**Response:** We have added the statement: "The funders had no role in study design, data collection and analysis, decision to publish, or preparation of the manuscript."

**Editor #**5. Please confirm that this does not alter your adherence to all PLOS ONE policies on sharing data and materials, by including the following statement:

**Response:** We have inserted the statement in Competing Interests: "This does not alter our adherence to PLOS ONE policies on sharing data and materials.”

**Editor #**6. Please include captions for your Supporting Information files at the end of your manuscript, and update any in-text citations to match accordingly.

**Response:** We have included the Supporting Information section with appropriate titles.

**Editor #7:** While revising your submission, please upload your figure files to the Preflight Analysis and Conversion Engine (PACE) digital diagnostic tool, <https://pacev2.apexcovantage.com/>.

**Response:** Completed. This was very smooth, one of the nicest tools I’ve seen, and the Figures are now compliant.

**Reviewers' comments:**

**Concern #1:** The work is not well-grounded in the literature. Running the query “Covid-19 AND Netosis” returns 69 articles, which include several which are of direct relevance and are not currently cited [e.g. 34344929]. “Covid-19 AND Neutrophils AND Elastase” returns 36 articles. Some important work investigating the role of neutrophils in viral respiratory infection and association with severity is not discussed either [e.g. 29777224].

**Response:** True, this is becoming a large field and we struggled to summarize it concisely. We have added the 2 specific citations above, as well as certain other relevant new citations.

**Concern #2:** - The number of subjects used in comparisons is often very small, which inevitably undermines some of the conclusions. There is probably not much to be done at this stage (unless available public provided opportunities for re-use / independent validation). But it would be preferrable not to subdivide already small study groups (e.g in some instances, n=2, n=4, n=5, n=7 etc…). Just for context, this study, which is not yet publish but seeks to address similar questions comprises > 300 subjects. Given the extent of inter-individual variability in patient cohorts it is the kind of numbers that would permit to bring more convincing answers. In any case this limitation should at least be discussed.

**Response:** This is a reasonable concern. The recruitment of patients in the COVID-19 ICU was challenging for several reasons, but surprisingly, we captured a fairly high percentage of available cases (n=38). Fortunately for the community, but unfortunately for our study, the introduction of the vaccines, combined with early and more effective treatments, meant far fewer ICU cases as our studies progressed, especially along the development of the NET/elastase assay. Thus, the number of patients that we could obtain NE data was more modest (n=14). The smaller Ns stem from the number of those patients that also received vasopressors, and/or had available lactate or NLR. We did consider seeking biobank specimens, but none would have been amenable to the isolation of viable neutrophils for the NE kinetic assay, which essentially must be done fresh within an hour of collection. We are validating this potential POC assay in newer cohorts with a broader range of viral infections. As requested, we have noted the sample size as a new section “Limitations” in the Discussion.

**Concern #3:** - The transcriptome analysis was very cursory. It seems the authors had decided from the onset to focus on neutrophils markers. The paper would benefit from a more global analysis being performed before deep diving on neutrophil related genes and neutrophils.

**Response:** We tried to be very transparent about the fact that we had previously identified these RNA biomarkers by whole transcriptome analysis of lower respiratory infections, and so we were curious whether they worked in COVID19. But, to remain open to new or better markers, we also conducted the RNAseq transcriptome analysis. Fortunately, the RNAseq and ddPCR confirm the strong movement of these biomarkers, and the RNAseq does identify other biomarkers that could be used. For instance, based on our, and others profiling studies, we incorporated myeloperoxidase (MPO) and resistin (RSTN) into our ddPCR analysis, but we do not see an obvious improvement in the predictive ability, and their overall levels are much lower. Also, we actually did a very deep analysis of the transcriptome data, including compiling and annotating novel sets of cell specific markers for the Illumina platform, as well as doing literature searching and further annotation on hundreds of differentially expressed genes. We tried to present the results in a relatively digestible format for clinicians and other healthcare professionals, but that may have omitted some of the more typical ‘heatmap’ clustering figures that may have been expected by genomics specialists.

To address this issue directly, we have included 3 new analyses: one which looked at the DEGs as a function of viral RNAemia by ddPCR (S3 Table A), another which analyzes the DEG transcripts using Ingenuity Pathway Analysis (Figure 1, Panel B), and a third which analyzes the DEGs using gene ontology analysis (S2B, S2C Tables). The RNAemia analysis contains a detailed manual curation of ~200 transcripts in the supplementary data. Combined, they further strengthen the evidence for strong neutrophil/NET-related activation, and T cell suppression of the immune system by SARS-CoV2.

**Concern #4:** - The quality of the figures is generally not very good, and they are not always informative. The DAPI staining images are not very clear and the labels that are overlaid obstruct a good part of the image. The use of an arrow on the line graph on Figure 9 is somewhat unusual for an original research paper. There are currently 9 figures, and the paper would likely benefit from focusing on those that are considered more critical to the message being conveyed.

**Response:** We apologize for this, it seems likely they may have been adversely affected by upload and compressions. Our versions of the DAPI staining, especially Figure 6, are very crisp. Possible the problem arises in Figure 7, where after the 30 minute incubation on slide the formation of NETs make that image quite ‘fuzzy’, but that is actually correct. We have brightened the image to make the NETs more obvious. We have moved the labels covering the DAPI in Figure 6, removed the arrow in Figure 9, and re-examined all the figures for clarity, using the PACE tool offered by PLOS One. High resolutions images, 330 DPI .tif, are provided in the upload, but the versions in the text remain .pdf to manage the size of the file.

**Concern #5:** - Before acceptance should be deposited in GEO and token provided for reviewer access (main point here would be to verify that the level of details provided is sufficient)

**Response:** Completed. The data is now uploaded to GEO under the accession #GSE189990 with all the relevant details necessary to reproduce our findings.
